# Left atrial appendage thrombus formation in a patient with atrial fibrillation on dabigatran therapy associated with CES1 and ABCB1 genetic polymorphisms: A case report

**DOI:** 10.1097/MD.0000000000022084

**Published:** 2020-09-04

**Authors:** Tingting Wu, Xiaotong Xia, Jinglan Fu, Wenjun Chen, Jinhua Zhang

**Affiliations:** a Department of Pharmacy, Fujian Medical University Union Hospital; b College of Pharmacy, Fujian Medical University, Fuzhou, Fujian, China.

**Keywords:** *ABCB1*, *CES1*, dabigatran, genetic polymorphisms, left atrial appendage thrombus

## Abstract

**Rationale:**

Dabigatran is a direct thrombin inhibitor that is widely used to prevent the formation of thrombus formation. Amiodarone can increase the plasma concentration of dabigatran. *CES1* (carboxylesterase 1) and *ABCB1* (ATP-binding cassette subfamily B member 1) genetic polymorphisms associate with the pharmacokinetics of dabigatran.

**Patient concerns:**

A 62-year-old woman was admitted to the hospital due to chest tightness, fatigue, and discomfort despite long-term anticoagulation with dabigatran 110 mg twice daily for 6 months, with concomitant use of amiodarone.

**Diagnoses:**

Left atrial appendage thrombus formation with a history of atrial fibrillation.

**Interventions:**

The clinician changed dabigatran to warfarin. To explore the causes of insufficient anticoagulation using dabigatran in this patient, we examined the *ABCB1* and *CES1* genes. Results showed that she carried *ABCB1* variant alleles with 3 heterozygote single nucleotide polymorphisms (SNPs: rs4148738, rs1045642, rs2032582) and *CES1* variant alleles with 2 heterozygote SNPs (rs2244613, rs4580160).

**Outcomes:**

The left atrial appendage thrombus disappeared.

**Lessons:**

Multiple mutations in the *ABCB1* and *CES1* genes may influence the pharmacokinetics of dabigatran and could have contributed to the thrombus formation in the left atrial appendage.

## 1 Introduction

Atrial fibrillation (AF) is the most common arrhythmia, with an incidence of approximately 2% to 3% in the general population,^[1]^ increasing the risk of ischemic stroke by about 5 times.^[2]^ In recent years, the safety and efficacy of catheter ablation for the treatment of symptomatic AF has been clinically verified and confirmed, especially for those patients who cannot tolerate anti-arrhythmia drugs or have shown no efficacy after treatment with these drugs.^[3]^ However, left atrial thrombus (LAT)/left atrial appendage thrombus (LAAT) is a contraindication for catheter ablation. Effective anticoagulation should be performed for at least 3 weeks before catheter ablation, and the presence of LAT and LAAT should be excluded before catheter ablation.^[4]^

Before catheter ablation, vitamin K antagonists (VKAs), or novel oral anticoagulants (NOACs) can be selected for oral anticoagulant therapy. The advantages of NOACs include a wide therapeutic window,^[5,6]^ no food interactions, few drug interactions, predictable pharmacokinetic and pharmacodynamic profiles and no coagulation monitoring compared with VKAs. NOACs are comparable to or even better than VKAs, with reduced overall mortality and cardiovascular mortality, lower incidence of intracranial hemorrhage and total bleeding^[7]^ and are increasingly used to prevent thrombosis. Dabigatran etexilate, the prodrug of dabigatran, is converted by carboxylesterase 1 (*CES1*) to the active form, dabigatran.^[8]^ In addition, dabigatran etexilate is the substrate for P-glycoprotein (P-gp), which is an efflux transporter. The ATP-binding cassette subfamily B member 1 (*ABCB1*) gene encodes P-gp.^[9]^ Some studies have shown that individual differences exist in clinical dabigatran etexilate anticoagulation therapy, which were associated with polymorphisms in the *CES1*- and *ABCB1*-related genes.^[10]^ A total of 80% of dabigatran is excreted by the kidneys, and its half-life is related to the patients renal function and creatinine clearance rate. Renal function can affect the elimination of dabigatran and, thus, affect its efficacy, therefore it is important to pay close attention to the renal function of patients.^[11]^ Dabigatran drug interactions are primarily mediated by P-gp rather than cytochrome P450,^[12]^ so caution should be exercised during the concomitant use of P-gp inducers, such as rifampicin (decreased concentration), and P-gp inhibitors, such as ketoconazole, quinidine, amiodarone, and verapamil (increased concentration).^[13,14]^ Herein, we report a patient with AF that was treated with dabigatran 110 mg twice daily for 6 months prior to catheter ablation. The patient developed LAAT due to insufficient anticoagulation; we analyzed the causes of LAAT formation under this antithrombotic condition.

## 2 Case report

**Figure 1:**
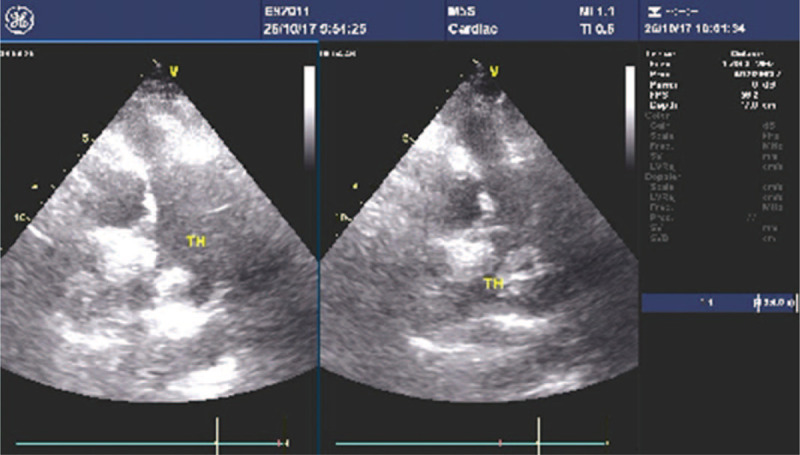
Echocardiography showing a large thrombus in the left atrial appendage after 6 months of anticoagulation with dabigatran.

**Figure 2:**
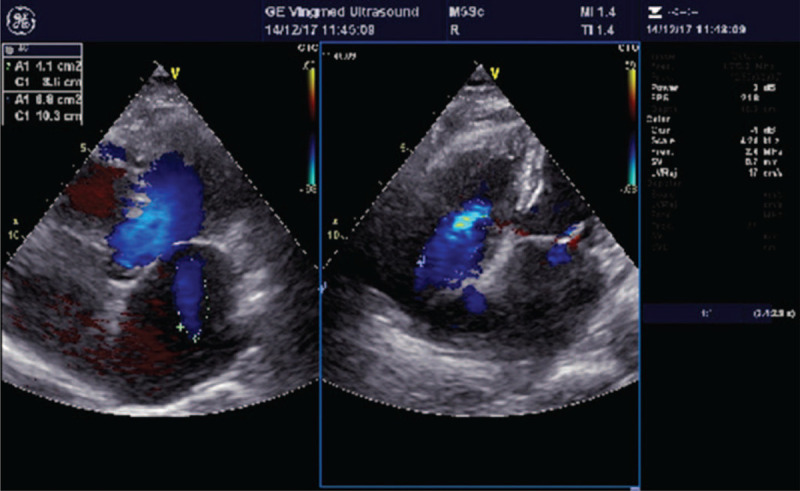
Echocardiography showing no thrombus in the left atrial appendage after 50 days of anticoagulation with warfarin.

The patient, a 62-year-old female, was admitted to the local hospital on May 23, 2016 due to chest tightness, palpitation and fatigue for 2 days. She was diagnosed with “AF, dilated cardiomyopathy, grade 2 cardiac function and thalassemia”, and the symptoms improved after drug treatment, including warfarin anticoagulant treatment. The patient was regularly monitored for international normalized ratio (INR), which was basically stable within the target range. On November 8, 2016, the patient had a echocardiography examination, showing that there was no thrombosis. On April 20, 2017, it was changed to receive 110 mg of dabigatran twice daily to prevent stroke and systemic embolism. Along with the administration of dabigatran, the patient received digoxin (0.125 mg qd), furosemide (10 mg qd), spironolactone (20 mg qd), bisoprolol (2.5 mg qd), amiodarone (100 mg qd), and perindopril (4 mg qd). The patient had good compliance. On October 26, 2017, the patient was readmitted to the hospital due to chest tightness, fatigue and discomfort. She decided to receive catheter ablation, and the echocardiography examination showed a large thrombus (31 mm × 27 mm) in the left atrial appendage (Fig. 1), with an enlarged left atrium diameter (57 mm) and normal cardiac ejection fraction (66%). The catheter ablation was cancelled because of the left atrial appendage thrombus. The clinician changed dabigatran to 2.5 mg warfarin daily and continued to give the patient bisoprolol to control the ventricular rate. The patient did not complain about heart palpitations, chest tightness, or other discomforts and was discharged. INR was monitored regularly after discharge to control INR within the target range. On December 14, 2017, the patient underwent another echocardiography, and the left atrial appendage thrombus had disappeared (Fig. 2).

The patients renal function was normal, with 51.5 μmol/L of creatinine. The creatinine clearance rate was 81 ml/minute, and the glomerular filtration rate was 136 ml/minute/1.73 m^2^. The medications the patient was taking included perindopril, bisoprolol, digoxin and amiodarone, among which, amiodarone is a moderate P-gp inhibitor that can increase the plasma concentration of dabigatran.^[15]^ Other drugs have not been reported to have an effect on the metabolism of dabigatran. It has been reported that the *ABCB1* and *CES1* genes are involved in the pharmacokinetics of dabigatran. To explore the causes of insufficient anticoagulation using dabigatran in this patient, we examined the *ABCB1* and *CES1* genes. The polymorphisms of *ABCB1* (rs 4148738, rs1045642, rs2032582), *CES1* (rs 8192935, rs 2244613, rs71647871), and *CES1P2* (rs 4580160) were determined by polymerase chain reaction product sequencing. The polymerase chain reaction primers are presented in Table 1. The results showed that the patient exhibited mutations at all 3 sites of *ABCB1*, but only 2 sites of *CES1* (rs2244613 and rs4580160). All site mutations were heterozygous mutations, as shown in Table 2.

**Table 1:**
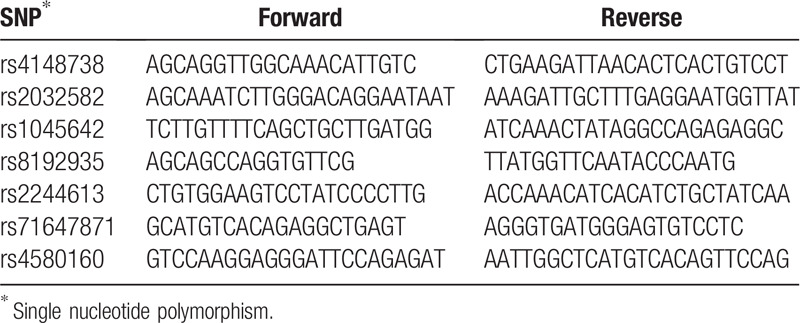
The polymerase chain reaction primers used for amplification of ABCB1 and CES1.

**Table 2:**
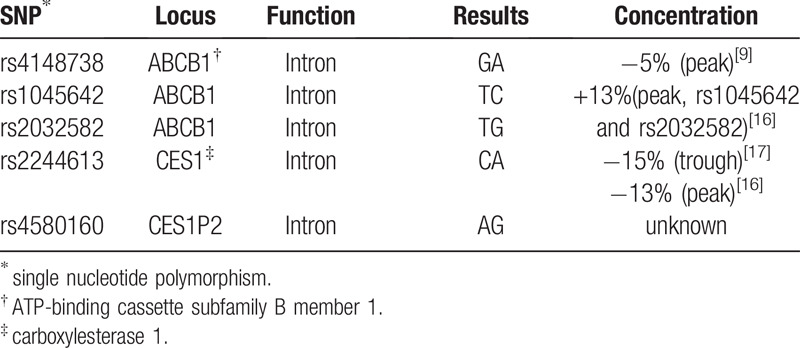
The results of ABCB1 and CES1 genotypingand the effects on the concentration of dabigatran according to previous studies.

## 3 Discussion

We have reported a case of AF with thrombus in the left atrial appendage despite receiving treatment with 110 mg of dabigatran twice daily for 6 months prior to catheter ablation. Our patient demonstrated normal renal function combined with the P-gp inhibitor, amiodarone. Amiodarone can increase the plasma concentration of dabigatran by 12% to 60% according to the 2018 European Heart Rhythm Association Practical Guide on the use of non-vitamin K antagonist oral anticoagulants in patients with AF.^[15]^ However, mutations in *ABCB1* and *CES1* alleles may affect dabigatran plasma concentration resulting in failure of dabigatran anticoagulation. Patients with AF carrying 1 or 2 A alleles showed 5% lower trough concentration compared with homozygotes for *ABCB1* single nucleotide polymorphisms (SNP) rs4148738 (G allele).^[9]^ Gouin-Thibault et al^[16]^ performed a haplotype combination analysis of *ABCB1* SNP rs2032582 and rs1045642 and found that homozygous and heterozygous mutations increased the peak plasma concentration (C_max_) of dabigatran by 33% and 13%, respectively, and the AUC by 28% and 25%, respectively. The *CES1* SNP rs2244613 is associated with a 15% decrease in adjusted trough concentration of dabigatran per minor allele.^[17]^ Gouin-Thibault et al^[16]^ indicated that heterozygous and homozygous *CES1* rs2244613 mutated groups showed 14% and 26% lower AUC values, respectively, as well as 13% and 43% lower C_max_ values, respectively, compared with the wild-type groups. However, no correlation has been reported between *CES1P2* SNP rs4580160 and plasma concentration or anticoagulant efficacy of dabigatran. The association between gene polymorphisms and ischemic events in patients taking dabigatran has not been reported. The patient we reported here had multiple gene site mutations in *ABCB1* and *CES1*. According to a previous study by Dimatteo et al,^[9]^ an *ABCB1* rs4148738 from GG to GA mutation may reduce the plasma concentration of dabigatran by 5%. According to the studies of Pare and Gouin-Thibault et al,^[16,17]^ a heterozygous mutation in *CES1* rs2244613 may reduce the trough concentration of dabigatran by 15% and the peak concentration by 13%, whereas the heterozygous mutation of *ABCB1* rs2032582 and rs1045642 may increase the peak concentration of dabigatran by 13%. We suspect that the ability of heterozygous mutations (*ABCB1* rs4148738 and *CES1* rs2244613) to down-regulate the plasma concentration of dabigatran is stronger than to up-regulate the plasma concentration of dabigatran due to additional factors, including *ABCB1* rs2032582 and rs1045642 heterozygous mutations and the combined use of amiodarone. The overall plasma concentration of dabigatran in the patient decreased, eventually leading to the formation of the left atrial appendage thrombus.

Currently, there have been very few cases of left atrial appendage thrombi forming in patients with AF treated with dabigatran. Zhi-Chun Gu et al^[18]^ reported a case of left atrial appendage thrombosis in a patient with persistent AF after anticoagulant treatment with 110 mg dabigatran twice daily for 31 months. The possible reason given by the authors was that the patient had multiple gene locus mutations in the *ABCB1* and *CES1* genes, and combined treatment with atorvastatin. In contrast, amiodarone, a moderate P-gp inhibitor, was administered in our patient reported here, which increased the plasma concentration of dabigatran by 12% to 60%,^[15]^ increasing the risk of bleeding. In addition, the patient we reported demonstrated heterozygous mutations, including *ABCB1* rs2032582 and rs1045642. However, the polymorphisms *ABCB1* rs2032582 and rs1045642 were not observed in the case report by Zhi-Chun Gu et al.

Our report has a few potential limitations. First, we were unable to determine the actual plasma concentration of dabigatran in the patient at the time, and the speculation of plasma concentration caused by gene mutation was based on the reported studies related to the pharmacogenomics of dabigatran. Secondly, the pharmacogenomics of dabigatran is a relatively new field; there are still many gene polymorphisms to be examine, therefore it is possible that other potential unknown gene polymorphisms have influenced the plasma concentration of dabigatran.

## 4 Conclusion

In the present case, we suggest that mutations in the *ABCB1* and *CES1* genes may be the main cause of dabigatran anticoagulation failure. Under the combined use of amiodarone, we suspect that the ability of heterozygous mutations (*ABCB1* rs4148738 and *CES1* rs2244613) to down-regulate the plasma concentration of dabigatran is stronger than to up-regulate the plasma concentration of dabigatran due to additional factors, including *ABCB1* rs2032582 and rs1045642 heterozygous mutations and the combined use of amiodarone. The overall plasma concentration of dabigatran decreased in the patient, eventually leading to thrombus formation in the left atrial appendage. In addition, although no related studies have been reported that show the relationship between *CES1P2* rs4580160 and dabigatran plasma concentration, the heterozygous mutation of this gene locus may affect dabigatran plasma concentration. Therefore, more clinical studies are needed to clarify the effects of *ABCB1* and *CES1* gene polymorphisms on the pharmacokinetics of dabigatran and thrombus formation in the left atrial appendage.

## Author contributions

**Conceptualization:** Tingting Wu, Jinhua Zhang.

**Data curation:** Jinglan Fu, Wenjun Chen.

**Formal analysis:** Xiaotong Xia, Tingting Wu.

**Project administration:** Jinhua Zhang, Tingting Wu.

**Writing – original draft:** Tingting Wu.

**Writing – review & editing:** Jinhua Zhang, Xiaotong Xia, Jinglan Fu, Wenjun Chen.
